# A Rare Case of Vertical Patella Dislocation Reduced by Closed Reduction

**DOI:** 10.7759/cureus.44756

**Published:** 2023-09-05

**Authors:** Temple Ihedinmah, Lauren Paish

**Affiliations:** 1 Emergency Medicine, Wyckoff Heights Medical Center, Brooklyn, USA

**Keywords:** emergency room patella reduction, closed reduction of patella, open reduction of patella, patella reduction, vertical patella dislocation, patella dislocation, locked patella dislocation

## Abstract

Vertical patella dislocation or “dorsal fin patella” is a rare but potentially debilitating orthopedic condition characterized by the displacement of the kneecap in a vertical direction. It occurs when the patella moves out of its normal position within the trochlear groove of the femur, rotating along its vertical axis resulting in the articular surface facing either medially or laterally. The mechanism of injury is primarily through axial rotation injury at the knee but could also occur secondary to a medial or lateral trauma to the patella. This dislocation can cause significant pain, immobility, and instability of the knee joint. Closed reduction to restore the patella to its proper alignment is a non-surgical technique that is uncommonly employed to treat vertical patella dislocations due to the inherent risk of osteochondral lesions.

Here, we present a case of a 39-year-old male with a vertical patella dislocation after sustaining a traumatic blow to the knee during a Jiu-jitsu sparring exercise with a partner. This injury was successfully managed by a closed reduction in the emergency department.

## Introduction

Visits to the emergency department due to patella dislocation are very common, with an annual incidence of 5.8 per 100,000 individuals, with lateral patella dislocation by far the most common [1]. The incidence increases with activities that either involve motions exposing the knee to excessive torque or direct traumatic blows to the patella [2]. The condition is commonly seen among young patients aged 10-17 years, where the incidence increases to 29 per 100,000 [3]. Patella rotation along its longitudinal axis with the articular surface facing laterally or medially is referred to as a vertical dislocation. When the rotated patella is locked inside the femoral groove, the dislocation is intra-articular; however, when the dislocated patella is wedged against the condyle, typically the lateral side of the lateral condyle, it is extra-articular. The patellar vertical dislocation that occurs extra-articularly is uncommon. To our knowledge, relatively few cases have been reported since it was first described by Sir Astley Cooper [4].

Patients with acute patellar dislocations are frequently in significant pain. If the patella does not reposition itself spontaneously, closed reduction with pressure is applied anteromedially on the lateral patellar edge during the deformity. This technique aids in the separation of the patella from the femoral condyles. With a moderate push on the lower pole of the patella, the patella instantly returns to its natural position. It takes fewer than 30 seconds to complete the maneuver. To avoid re-dislocation, a plaster-of-Paris slab is used to hold the knee in 20-degree flexion [5]. However, with vertical patella dislocation, the risk of osteochondral lesions makes surgery the first option over closed reduction for the safe reduction of the patella [6].

## Case presentation

A 39-year-old athletic male with no known medical history presented to the emergency department following a Jiu-jitsu training session with complaints of left knee pain and a grossly dislocated patella. He had no associated sensory or motor deficits in areas distal to the injury. The sparring partner placed his knee on the patient's thigh; he slid his knee down toward the patient's toes. The patient subsequently heard a “popping” sound accompanied by intense pain and was unable to bear weight on the left lower extremity immediately afterward.

**Figure 1:**
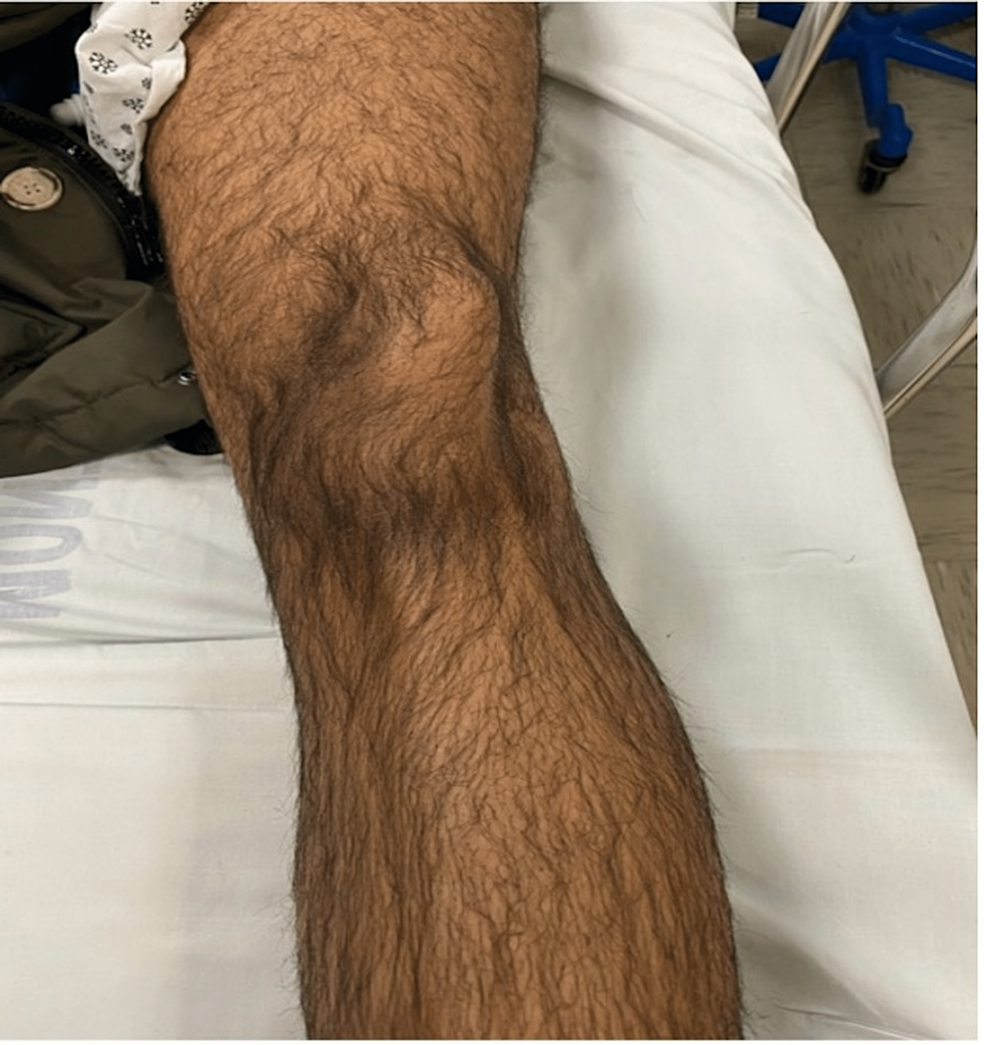
Lateral displacement of the patella as seen on presentation

**Figure 2:**
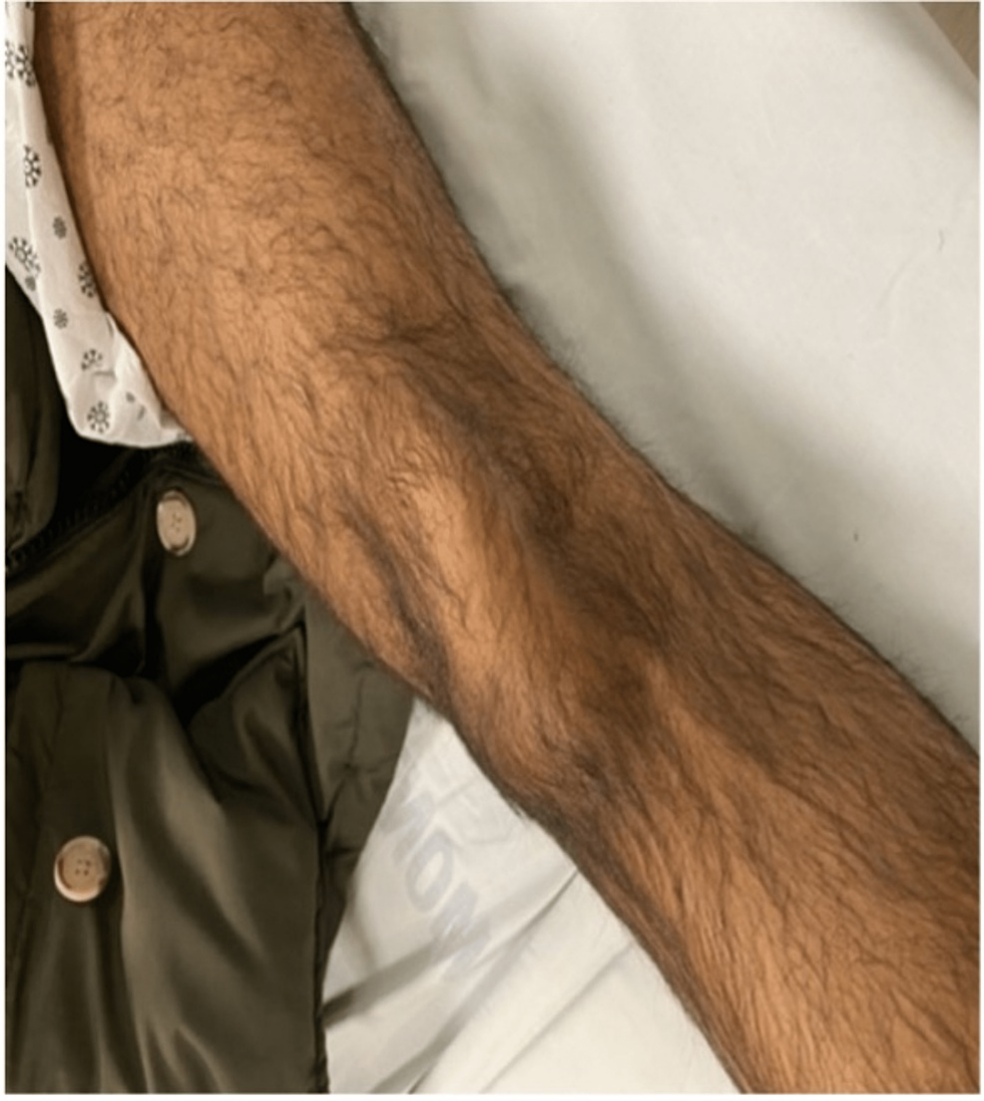
Another image showing lateral displacement of the patella

**Figure 3:**
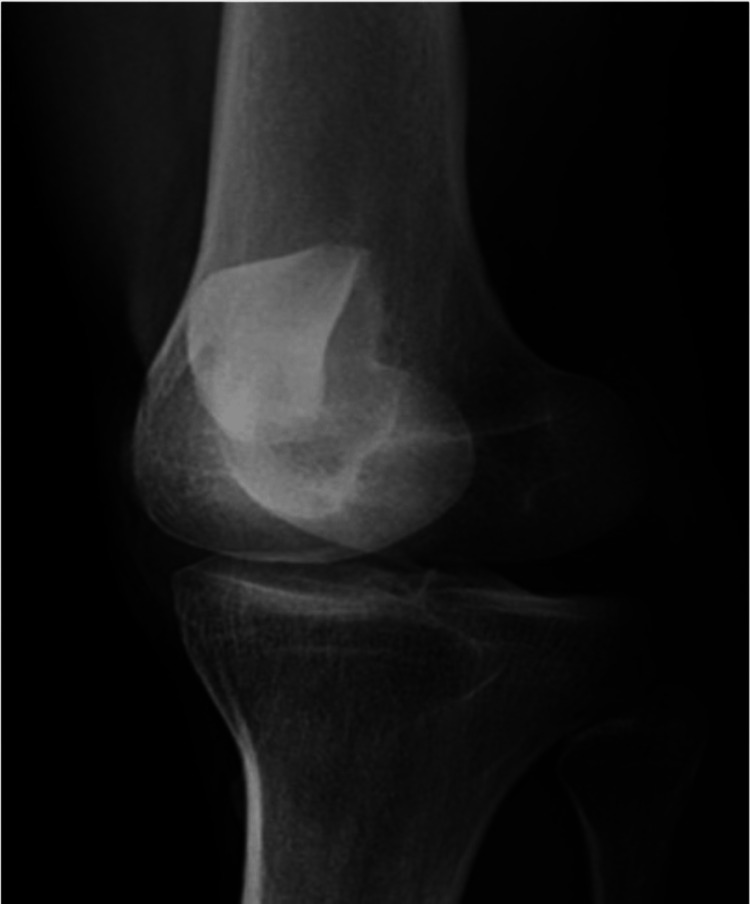
X-ray (anteroposterior view) showing the patella in an unusual position

**Figure 4:**
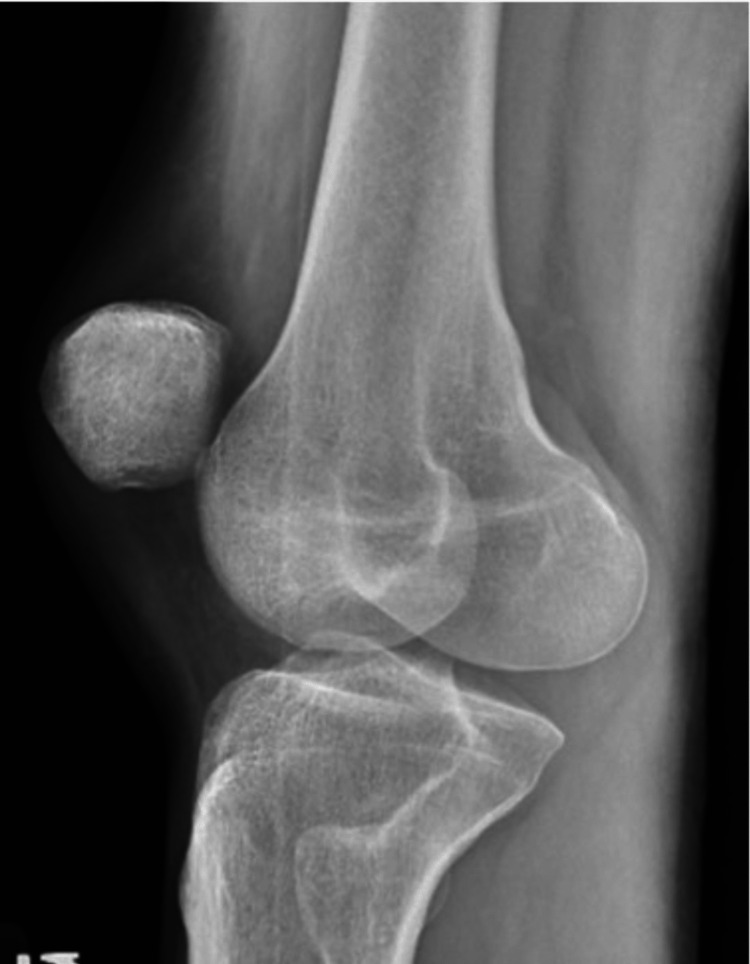
Lateral X-ray showing the patella in an unusual position

On presentation, the left knee was locked in full extension with the patella angulated at approximately 90 degrees vertically and the taut skin gave off a “dorsal fin” appearance (Figures 1, 2). Radiograph imaging showed an intra-articular vertically dislocated patella with the articular surface facing toward the medial aspect of the femoral condyle (Figures 3, 4). No fracture or other injury was noted on the X-ray.

**Figure 5:**
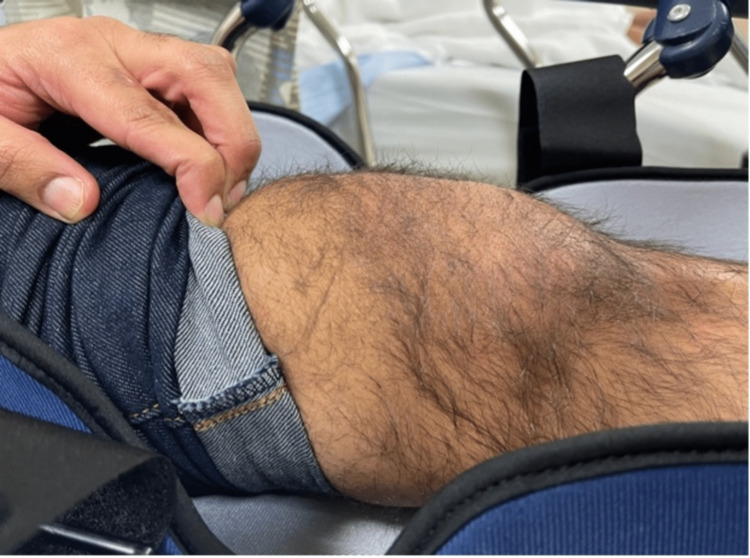
Reduction of the patella to its normal anatomic position

**Figure 6:**
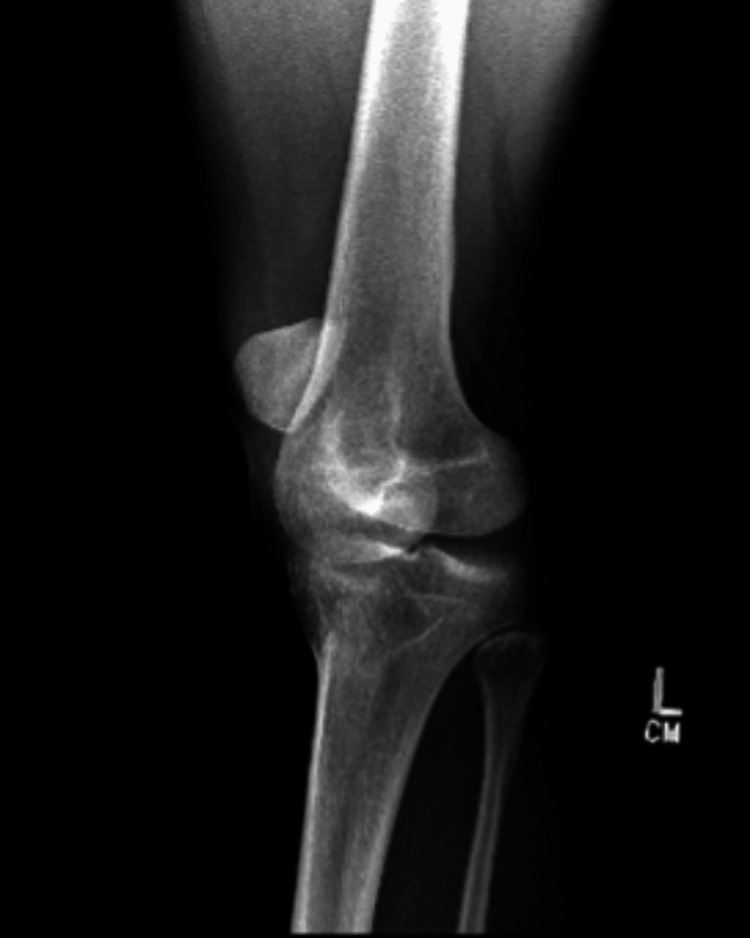
X-ray (oblique view) post-reduction

**Figure 7:**
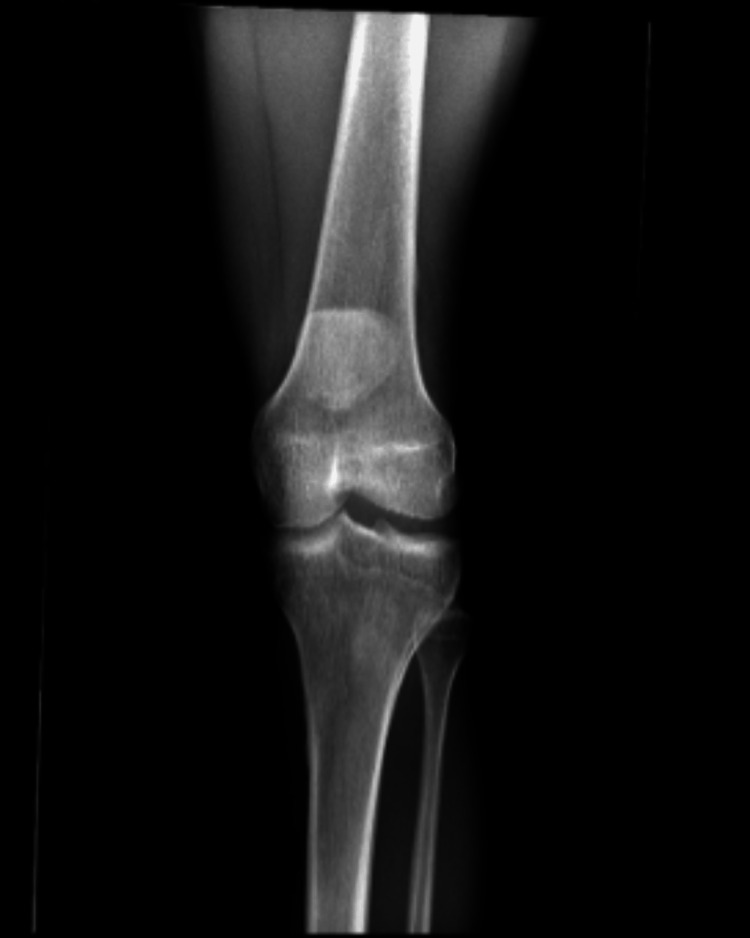
X-ray (anteroposterior view) post-reduction

Before patella reduction, intravenous morphine was administered to provide pain control through the procedure. While stabilizing the femur, pressure was applied to the patella from the lateral to the medial aspect of the knee resulting in the reduction of the patella to its normal anatomic position (Figure 5). A full range of motion of the knee was demonstrated immediately post-reduction. A post-reduction radiograph showed the proper anatomical alignment of the left patella (Figures 6, 7). A knee immobilizer and crutches were provided, and the patient was instructed to weight bear as tolerated on the left knee and to follow up with the orthopedic outpatient clinic in three days. There was no post-reduction knee arthroscopy completed.

The patient subsequently underwent an outpatient MRI study that showed no acute tearing of the medial knee joint capsule or any other evidence of ligamentous injury, and received eight weeks of physical therapy. There was no further dislocation of the left patella noted during this period. At a three-month follow-up post-dislocation, the patient reported that he was back to his pre-injury self, with only rare “pinching” pain in the anterior area of his knee.

## Discussion

In part due to quadriceps muscle contraction, the natural anatomic tendency of the patella is to displace laterally, with possible disruption of the medial patellofemoral ligament and medial retinaculum [7]. Intra-articular dislocations of the patella are uncommon and involve either horizontal or vertical axis patella dislocation, with horizontal dislocation being the less common of the two. Vertical axis dislocation has two types where the articular surface of the patella is either laterally or medially oriented. In horizontal axis intra-articular patella dislocation, the articular surface is either oriented towards the tibial articular surface or facing proximally with a detached inferior pole from the patella tendon [8].

The current literature indicates that the majority of cases are traumatic in nature and frequently occur in patients when participating in sports with a forceful impact directed at the knee [5]. Most occurrences have been documented in younger patients, with the rationale being that ligamentous laxity allows for more patellar rotation, while similar lesions are posited to likely cause a tendinous rupture in adults [9].

Albeit most authors recommend an open reduction as the initial reduction technique due to multiple reported unsuccessful closed reductions and risk of resultant osteochondral lesions [9], our patient in the ED was able to have his vertical patella dislocation reduced with minimal discomfort, demonstrating the successful use of morphine without local or general anesthesia as an analgesic in a closed reduction approach.

## Conclusions

Vertical patella dislocation is an uncommon distressing condition that affects the stability and function of the knee joint. Closed reduction with adequate analgesia, as a non-surgical technique, offers a safe and effective means of restoring the patella to its normal position. By adhering to the principles of closed reduction and providing appropriate post-reduction care, ED physicians can alleviate pain, restore joint stability, and facilitate a quicker recovery for patients. Although the closed reduction technique is straightforward, a surgeon must be available to do an open reduction if necessary.
